# Evaluation of design parameters on the outcome of intrastromal ring surgery using biomechanical simulations

**DOI:** 10.1371/journal.pone.0311926

**Published:** 2024-12-05

**Authors:** Shima Bahramizadeh-Sajadi, Hamid Reza Katoozian, Miguel Angel Ariza-Gracia, Morteza Gholami, Alireza Baradaran-Rafii, Philippe Büchler

**Affiliations:** 1 ARTORG Center for Biomedical Engineering Research, University of Bern, Bern, Switzerland; 2 Department of Biomedical Engineering, Amirkabir University of Technology (Tehran Polytechnic), Tehran, Iran; 3 Department of Mechanical Engineering, Sharif University of Technology, Tehran, Iran; 4 Ophthalmic Research Center, Research Institute for Ophthalmology and Vision Science, Shahid Beheshti University of Medical Sciences, Tehran, Iran; 5 Department of Ophthalmology, Morsani College of Medicine, University of South Florida, Tampa, Florida, United States of America; Kamuzu University of Health Sciences, MALAWI

## Abstract

**Background:**

The cornea plays a role in the refractive power of the eye, and when its natural curvature and thickness are compromised by diseases such as keratoconus or high myopia, this results in loss of visual acuity. Intracorneal rings (ICRs) were developed as a treatment option to restore the natural corneal curvature by implanting rings into tunnels cut within the corneal stroma. However, selecting and placing the appropriate ring can be difficult, and predicting refractive outcomes is challenging.

**Objective:**

The purpose of this study was to better understand the design parameters of the rings that determine postoperative refractive and mechanical outcomes.

**Methods:**

We developed an automated finite element simulation pipeline for ICR implantation and tested 300 variations of 20 ICRs.

**Results:**

The outcome of ICR was dominated by the vertical size of the ring; 84% of the change in corneal curvature can be attributed to the vertical size of the ring, while only 13% were attributed to the detailed cross-sectional shape of the ring. However, the cross-sectional shape of the ring is limited to the change in axial length and contact pressure between the ring and the cornea. The horizontal dimension of the ring plays only a minor role in the postoperative outcome.

**Conclusion:**

These results support Keraring’s approach to ring scaling, in which only the vertical dimension of the ring is changed, while the horizontal dimension remains constant. Numerical models help to understand ICR outcomes, design implants, and personalize empirical nomograms to achieve more successful postoperative outcomes.

## Introduction

The cornea is responsible for about two-thirds of the refractive power of the eye [1]. Pathological deformations of the cornea caused by diseases such as keratoconus (KC) result in significant and irreversible vision loss, which severely affects the quality of life of patients [2, 3]. The emmetropic cornea has a uniform curvature and thickness within the optical zone, but in patients suffering from KC, the cornea is locally weakened, leading to a progressive thinning and steeping of the cornea that progress over time and result in a very large refractive error, irregular astigmatism, and severe myopia [4, 5].

Intracorneal implants (ICRs) have been developed to treat these patients and restore some of the lost visual acuity. In this technique, one or two circular polymeric ring segments are implanted into a tunnel that is cut in the periphery of the cornea at the posterior half of the corneal stromal depth. Implantation of the ring flattens the cornea, and has been shown to effectively alleviate or stop the manifestations KC by restoring a more normal corneal curvature [6]. It has also been reported that this surgical technique can delay or even prevent the need for keratoplasty [5, 7]. ICR is also used for other pathologic conditions that do not allow refractive surgery due to changes in corneal shape and thickness [8–11]. Therefore, it can correct both morphologic and visual parameters of the cornea [12, 13].

Selection of the appropriate ring and surgical parameters for each patient is critical for optimal refractive outcomes. These parameters are currently determined using nomogram tables based on empirical observations. Clinical outcomes are controversial, as final results are difficult to predict and often unacceptable [8, 10, 14, 15], with only 57% of patients achieving a postoperative refractive error of less than 0.5 D [16]. If refractive outcomes are unfavorable, revision surgery and additional procedures may be required [17]. If the combination of photochemical cross-linking (CXL) and ICR surgery fails, the last treatment option is corneal transplantation [2], which is not only invasive but also depends on the availability of donor tissue. With approximately 12.7 million patients worldwide waiting for donor corneas [18], it is important to ensure optimal KC management to avoid corneal transplantation.

Machine learning has been proposed as a method to improve implant selection [19–21], and some studies have shown more promising results in visual prediction than manufacturers’ nomograms [19]. However, this approach lacks the comprehensibility that mechanistic in-silico models provide. Mechanical finite element (FE) simulations have been used to better understand the mechanisms of postoperative ICR correction [8, 11, 22–25]. These numerical studies analyzed various parameters of the surgical procedure, such as the cross-sectional shapes of the implant [26], its dimensions [11, 15, 23–27], the comparison of insertion methods [11, 15, 24], or the insertion depth [23, 24]. However, the numerical studies to date have focused only on simulating some geometric features of the surgical ICR simulations, as developing a fully automated FE methodology is challenging [23].

The objective of this study was to develop an automated finite element simulation pipeline for ICR implantation to better understand the design parameters of the rings that are critical for refractive outcome. We hypothesize that some geometric features of the ring can be used as predictors of surgical outcome, independent of the exact cross-sectional design of the ICR. Therefore, not only three commercial ring designs were studied, but a total of 20 cross-sectional designs with different implantation diameters, sizes, and shapes.

## Materials and methods

Numerical model of the implantation of ring were performed using the finite element method using the Abaqus Standard 2020 (Simulia, Dassault Systèmes, France).

### Model of the cornea

**Fig 1:**
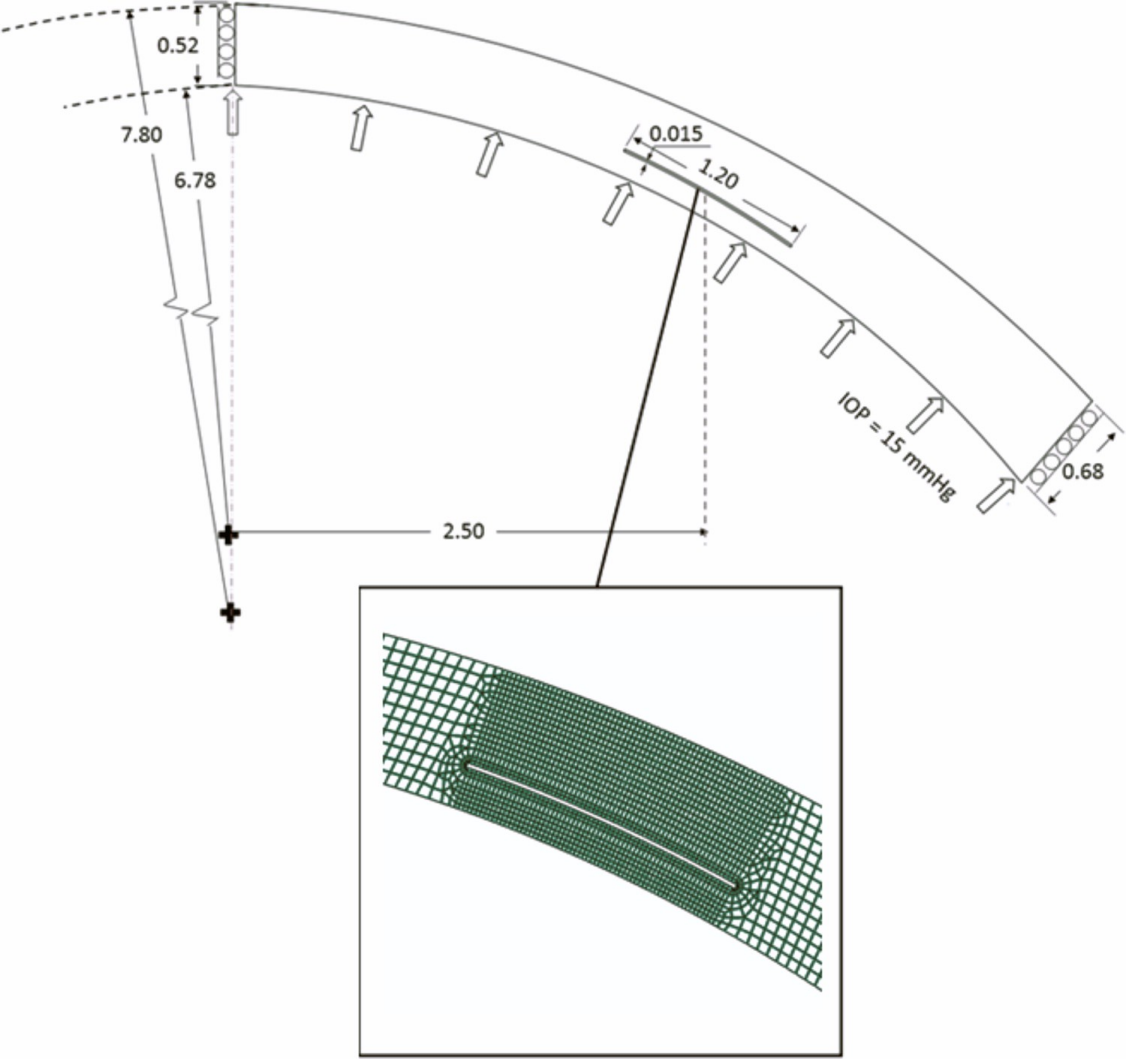
The axisymmetric model corresponds to an average human cornea (ref). The rings were implanted at the recommended depth corresponding to 75% of the corneal thickness and at three different implantation diameters of 5, 5.5, and 6 mm. This figure shows the anatomical dimensions of the corneal model and the generated tunnel as well as the refinement of the finite element mesh in the region of the implantation tunnel. All dimensions in the figure are in millimeters.

An axisymmetric model representing an average cornea was developed. The anterior and posterior curvatures of the cornea were 7.80 mm and 6.78 mm, respectively. The cornea was modeled with corneal thickness of 0.52 mm at the center and 0.68 mm at the corneoscleral border [23, 24]. Approximately 2900 quadrilateral elements were used to mesh the cornea (Fig 1).

An incompressible Yeoh material model was used to describe its mechanical behavior:

$$W=\sum_{i=1}^{3}C_{i}{\left(I_{1}-3\right)}^{i}$$

Where *I*_1_ is the first invariant of the right Cauchy-Green tensor, and *C*_1_ = 35.5 kPa, *C*_2_ = 3.2 kPa, *C*_3_ = 1.9 kPa are material parameters [11].

Prior to implantation of the rings, the stress-free geometry of the cornea was calculated using an iterative algorithm so that its shape matched the average shape described above under physiological IOP of 15 mmHg [28]. This procedure has already been described and is therefore only briefly explained here. A finite element mesh of the cornea in its physiological configuration is created. This model is loaded by an intraocular pressure of 15 mmHg, while the model is free to expand at the corneoscleral junctions on a plane inclined at 40 degrees [29]. For each node of the mesh, the distance between its position after application of the pressure and its position at the beginning of the simulation is subtracted from its initial position to obtain an updated corneal shape. This process is repeated until the distance between the position of the nodes after application of IOP corresponds to the desired physiological shape of the cornea with an error of less than 10^−2^ μm. This new corneal model is considered the stress-free geometry of the corneal model. Once the tension-free shape is achieved, the elements within the intended tunnel area were removed to simulate the formation of the tunnel within the tensioned cornea.

### Intracorneal rings

**Fig 2:**
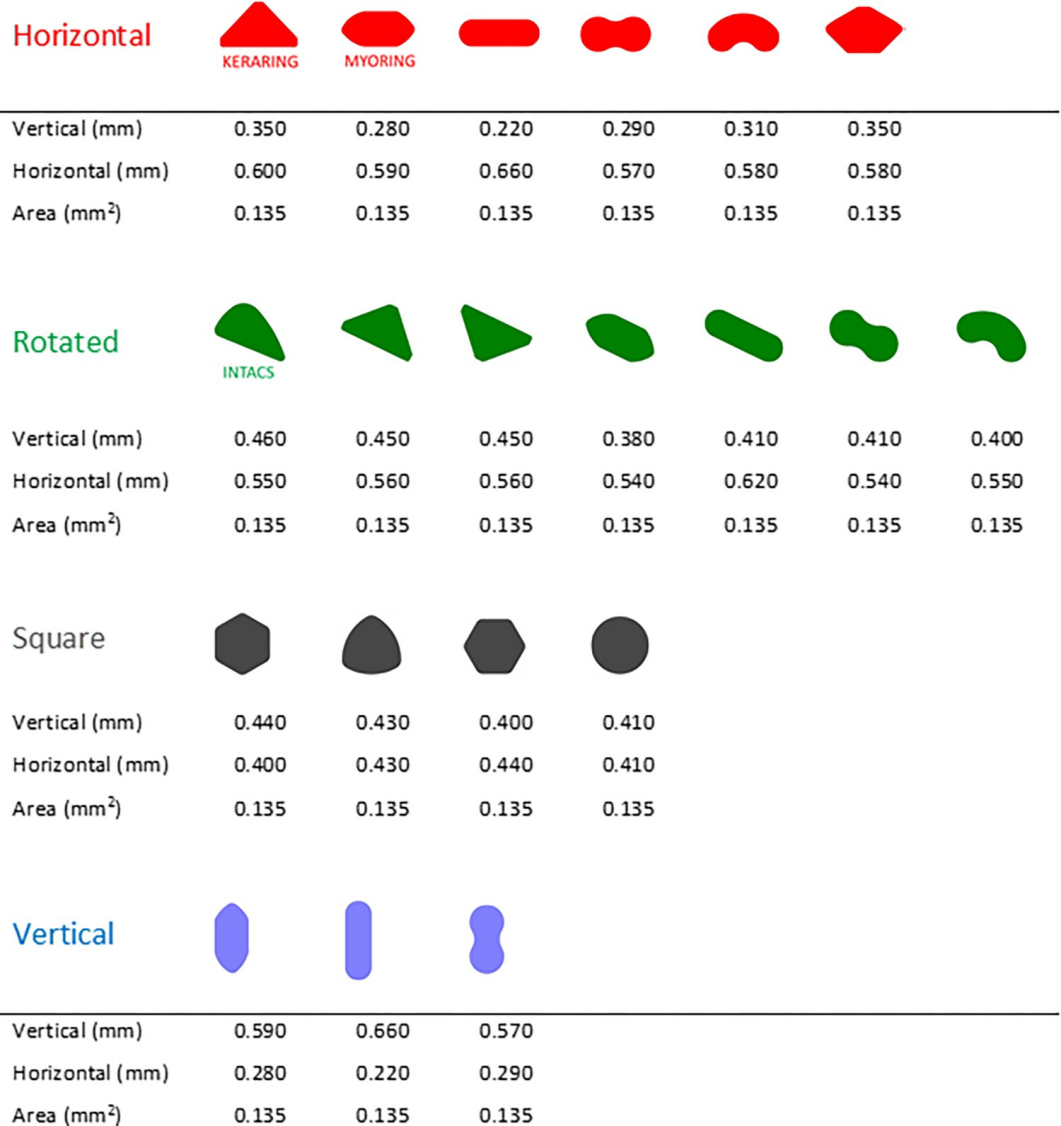
Design of the 20 ring families used in the study and their dimensions, categorized as horizontal, rotated, square, and vertical. The dimensions given here correspond to the largest of the 5 cross sections of the rings. The dimensions of the smaller cross sections can be obtained by multiplying the vertical and horizontal lengths by 0.935, 0.869, 0.793, and 0.715, respectively. These dimensions result in identical cross-sectional areas as the five sizes of the Keraring, although the scaling between the different sizes of the Keraring is not isotropic (Fig 3).

**Fig 3:**
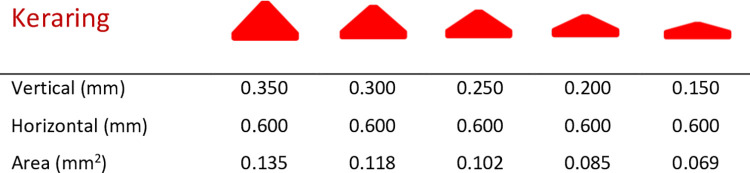
Geometric dimension of the Keraring. Unlike all other rings, the cross- sections for the different sizes of this implant are not determined by isotropic scaling of the base dimensions. In Keraring, the horizontal dimension remains constant across the different sizes and only the vertical dimension is changed.

In this study, we investigated various basic geometric features of the ring (Fig 2). Different rings were designed with different shapes, horizontal/vertical ratios, and orientations, while the cross-section remained identical to the basic profiles of the Keraring (Fig 3). A total of 20 ring designs with three diameters (D = 5.0 mm, 5.5 mm, and 6.0 mm) and five sizes each were considered, resulting in a total of 300 configurations. Among the proposed designs, several commercial deployments were considered: Keraring (Mediphacos, Belo Horizonte, Brazil), Intacs (Addition Technologies, Inc, Fremont, California, USA), and Myoring (Dioptex GmbH).

**Fig 4:**
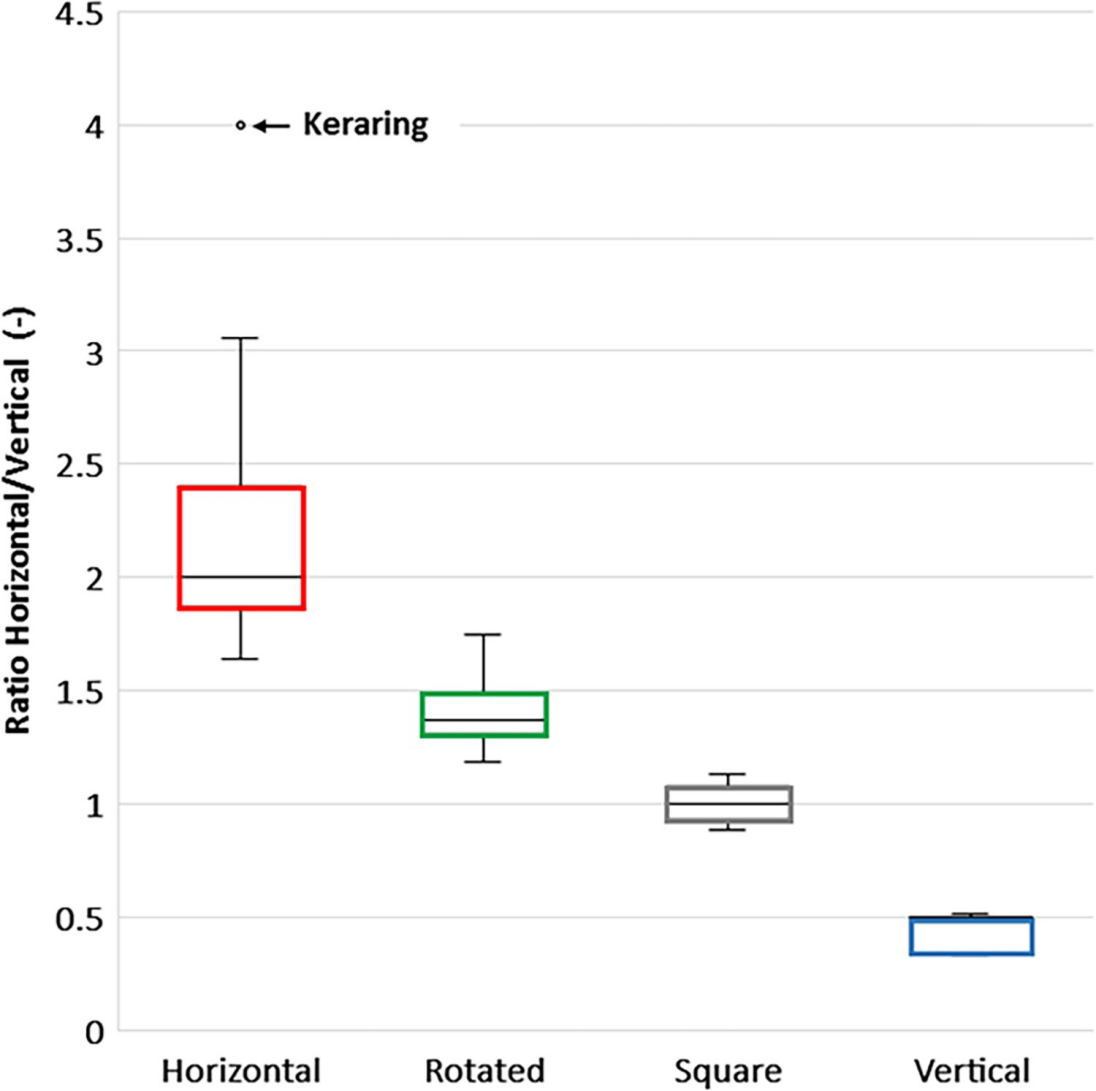
Each ring category can be distinguished by the ratio of horizontal and vertical ring dimensions.

The 20 rings investigated in this study were classified into four families based on the ratio between the horizontal and vertical dimensions of the ring cross-section (Fig 4). The horizontal family (ratio ≈ 2), the rotated family oriented in a direction parallel to the tunnel width (ratio ≈ 1.5), the square family with nearly equal length (ratio ≈ 1), and the vertical family (ratio ≈ 0.5).

Each of the rings was meshed with about 1600 linear quadrilateral elements and modeled as a linear elastic material with a Young’s modulus of 3600 MPa and a Poisson’s ratio of 0.4.

### In-silico ring implantation

**Fig 5:**
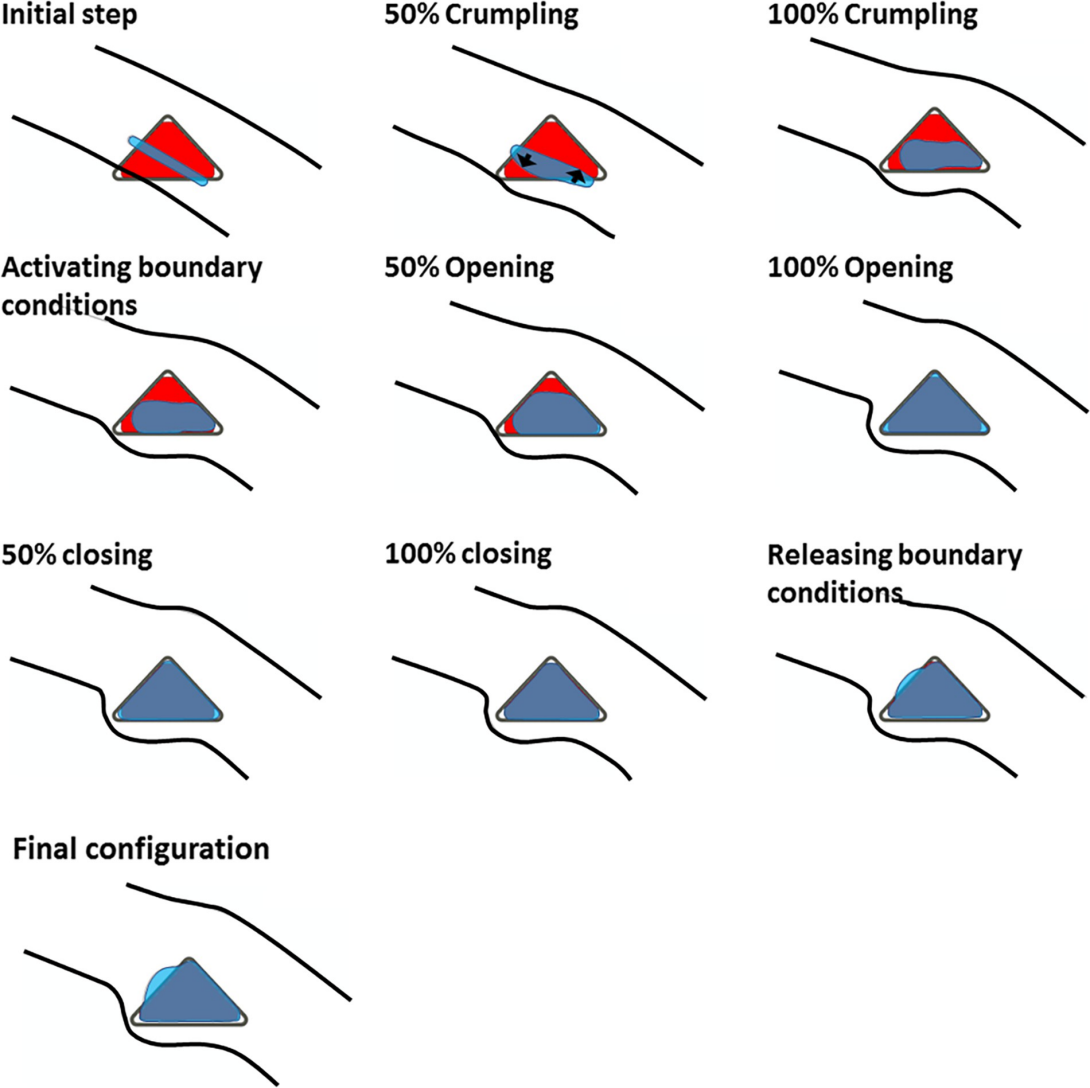
The ICR insertion steps are shown with the largest size Keraring (in red) in the corneal model (in black). The tunnel is shown in light blue, while the rigid tool that restricts the expansion of the ring is shown in dark grey. In the initial configuration, the tunnel extends beyond the rigid tool. Therefore, the simulation starts with a "crumpling step" in which a negative pressure is applied to two opposite sides of the tunnel to make it conform to the rigid surface (shown by arrows at "50% crumpling"). After crumpling, the contact between the tunnel and the rigid tool is activated and a pressure is applied to the inner surface of the tunnel. Expansion is limited by the rigid tool during inflation. At the end of inflation, the contact between the tunnel and the ring is activated and the pressure is reduced to zero, establishing contact between the corneal stroma and the ring. The center of mass of the rigid tool was located at the insertion depth of interest (i.e., 75% of the corneal thickness from the anterior surface).

The rings were implanted in a thin shallow slit of 15 × 1200 μm created at 75% of the corneal depth (Fig 1). The implantation tunnel created in the cornea is opened by applying a pressure load of 60 kPa (approximately 30 times the IOP) to the inner surfaces of the corresponding channel. To limit the opening of the tunnel and to avoid excessive deformation of the mesh and resulting convergence problems, the opening of the tunnel was limited by a rigid tool (Fig 5). The rigid tool was defined by an outwardly offset ICR cross-section with a small thickness, and a frictionless contact interaction was defined between the inner surface of the tunnel and this rigid tool. The rigid body is completely fixed from the beginning of the simulation. At the end of the expansion, a predefined contact interaction between the inner surface of the tunnel and the ring is reactivated, the pressure inside the tunnel is linearly released, and the corneal tunnel elements move backward until they reach the surface of the ring.

### Optical and statistical analysis

The results of the simulations were analyzed using optical parameters: average keratometry, change in axial length, and change in corneal central thickness.

The average keratometry K_mean_ was calculated over an optical zone of 3 mm in diameter of the anterior surface as follows [11]:

$$K_{mean}(D)=\frac{n-1}{R}$$

Where n is the index refraction of the cornea 1.3375, and R is the radius (in meters) of the best-fit sphere to the anterior surface around the corneal apex. The change in the axial length is affecting the refractive outcome of the intervention. It was calculated as the displacement of the node on the anterior surface of the cornea located on the symmetry axis. Finally, the change of corneal central thickness (CCT) was measured as the change in the distance between the two nodes on the symmetry axis located on the anterior and posterior surfaces, respectively.

The nonparametric Spearman method was used to evaluate possible correlations of change in K_mean_, axial length, corneal thickness, and contact pressure with horizontal and vertical dimensions. In addition, two-sided ANOVA and post-hoc Tukey’s Honest Significant Difference (HSD) were used to compare the means of intercategory differences among all treatment parameters.

To investigate which design parameters, contribute to the optical and mechanical outcomes in the corneal-ring relationship, multivariate regression using the backward elimination method of independent variables was performed between these outcomes and the basic geometric characteristics of the ring: horizontal and vertical dimension and implantation diameter, separately. Three different implantation diameters were considered: 5.0 mm, 5.5 mm and 6.0 mm. The predictive power of the basic geometric characteristics of the ring on the postoperative outcome was determined as follows: The multicollinearity of independent variables was assessed using the variance integration factor (VIF) to decide on the inclusion of basic parameters in the regression model. ANOVA was performed to determine which basic geometric parameters of the ring produced the most changes in the multivariate multiple regression analysis by adjusted coefficients of determination (R^2^_adj_) [30]. Statistical analysis was performed using RStudio.

## Results

**Fig 6:**
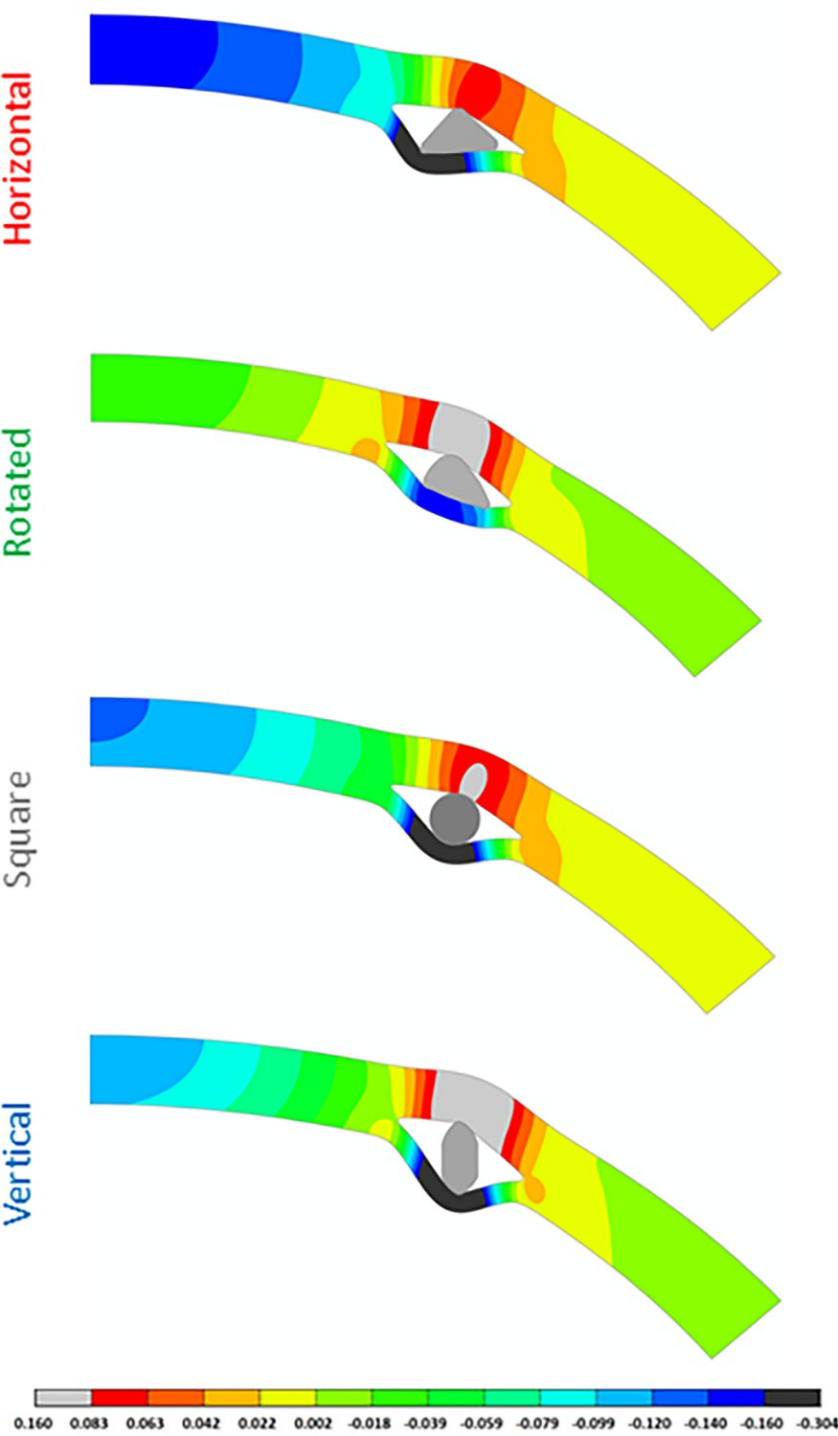
Displacement of the cornea along the optical axis with a representative ring for each category. In all cases, a negative displacement was observed in the apical region, corresponding to a flattening of the cornea, with this effect being more pronounced in horizontal and square rings. Large corneal displacements are also visible in the region of the ring where the stromal layer above the ring moves forward and the layer below the ring moves backward.

Five different dimensions were implanted for each ring design resulting in a total of 100 configurations. A flattening effect due to ICR implantation was observed for all ring categories. This flattening is associated with a negative shift of the apex and the surrounding central corneal area. This backward movement of the apex is visible for all ring types, but this movement is greater for horizontal and square rings (Fig 6). The ring also causes large local displacements and stresses in its immediate vicinity, but this stress concentration remains confined to the immediate vicinity of the ring and does not affect the overall stress pattern in the cornea. In all configurations, the ring causes stretching of the corneal layer anterior and posterior to the ring, resulting in a gap immediately medial and lateral to the ring. The vertical rings caused a larger gap than the rings in the rotated category due to their respective positioning.

**Fig 7:**
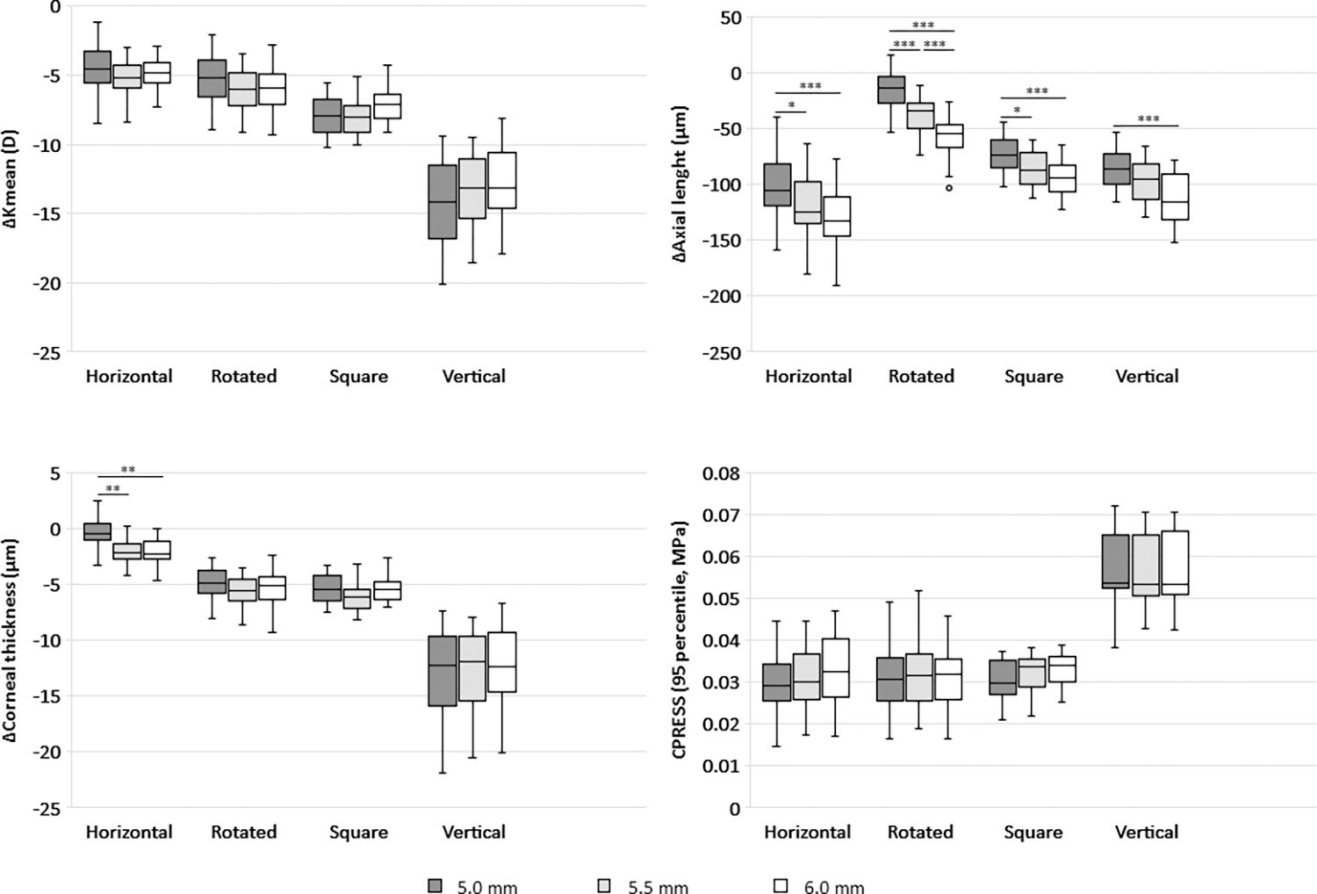
The change in K_mean_ (a), axial length (b), and corneal thickness (c) caused by the rings arranged by category and diameter. Vertical rings show the greatest change in K-mean and corneal thickness, whereas horizontal rings have the greatest effect on axial length. While the different types of rings exert similar pressure on the tunnel surface, the vertical rings are associated with significantly higher stresses (d). The darker the box color, the smaller optical zone diameter, or the ring is closer to the corneal center. All p values less than 0.001 are marked with three asterisks (***), those less than 0.01 are marked with two asterisks (**), and those less than 0.05 are marked with one asterisk (*).

The impact of ring category on key clinical outcomes was assessed (Fig 7). The results showed a significant difference in the induced change in K_mean_, axial length, and corneal thickness for all categories (p < 0.001), except for the change in axial length between square and vertical (p = 0.013) and for CCT between rotated and square (p = 0.814). All rings show a reduction in K_mean_, indicating the desired flattening of the cornea. Axial length is also reduced for almost all the rings, which also contributes to the desired optical correction by ring implantation. However, the interaction of this result with the change in corneal thickness shows that the reduction in axial length is achieved differently in the different categories: The horizontal rings cause an inward rotation of the central cornea, whereas the vertical rings are additionally associated with a significant thinning of the central cornea (Fig 7). The pressure exerted by the ring on the tunnel was also compared between the different categories (Fig 7). No difference in the maximum stress could be detected between horizontal, rotated, and square ring. The vertical rings were associated with significantly greater contact stress than the other ring categories (p < 0.001) but had a smaller contact area.

Overall, the ring diameters had less influence on the measured results than the ring aspect ratio and thus the ring family (Fig 7 and Table 1). When each ring family is analyzed independently, diameter has no effect on K_mean_ (p > 0.061), contact pressure (p > 0.262), and corneal thickness (p > 0.09), except for the horizontal ring family, where there is a significant difference between the smallest diameter and the larger ones (p < 0.002). However, the larger ring diameters significantly reduce the calculated axial length for all families (p < 0.027).

**Table 1:** P-values of the multiple comparison of the effects of ring’s diameter on the optical and mechanical post-implantation outcome. Tukey’s Honest Significant Difference (HSD) test is used as the post hoc test.

Parent family	Diameters (mm)	ΔK_mean_	ΔAxial length	ΔCCT	CPRESS
**Horizontal**	**5.0–5.5**	0.243	**0.031**	**<0.002**	0.850
	**5.0–6.0**	0.833	**<0.001**	**<0.002**	0.566
	**5.5–6.0**	0.551	0.289	0.999	0.882
**Rotated**	**5.0–5.5**	0.076	**<0.001**	0.113	0.934
	**5.0–6.0**	0.061	**<0.001**	0.557	0.921
	**5.5–6.0**	0.994	**<0.001**	0.587	0.999
**Square**	**5.0–5.5**	0.952	**0.027**	0.075	0.674
	**5.0–6.0**	0.263	**<0.001**	0.996	0.262
	**5.5–6.0**	0.155	0.356	0.09	0.746
**Vertical**	**5.0–5.5**	0.666	0.326	0.989	0.985
	**5.0–6.0**	0.354	**0.002**	0.914	0.983
	**5.5–6.0**	0.858	0.072	0.962	0.939

**Fig 8:**
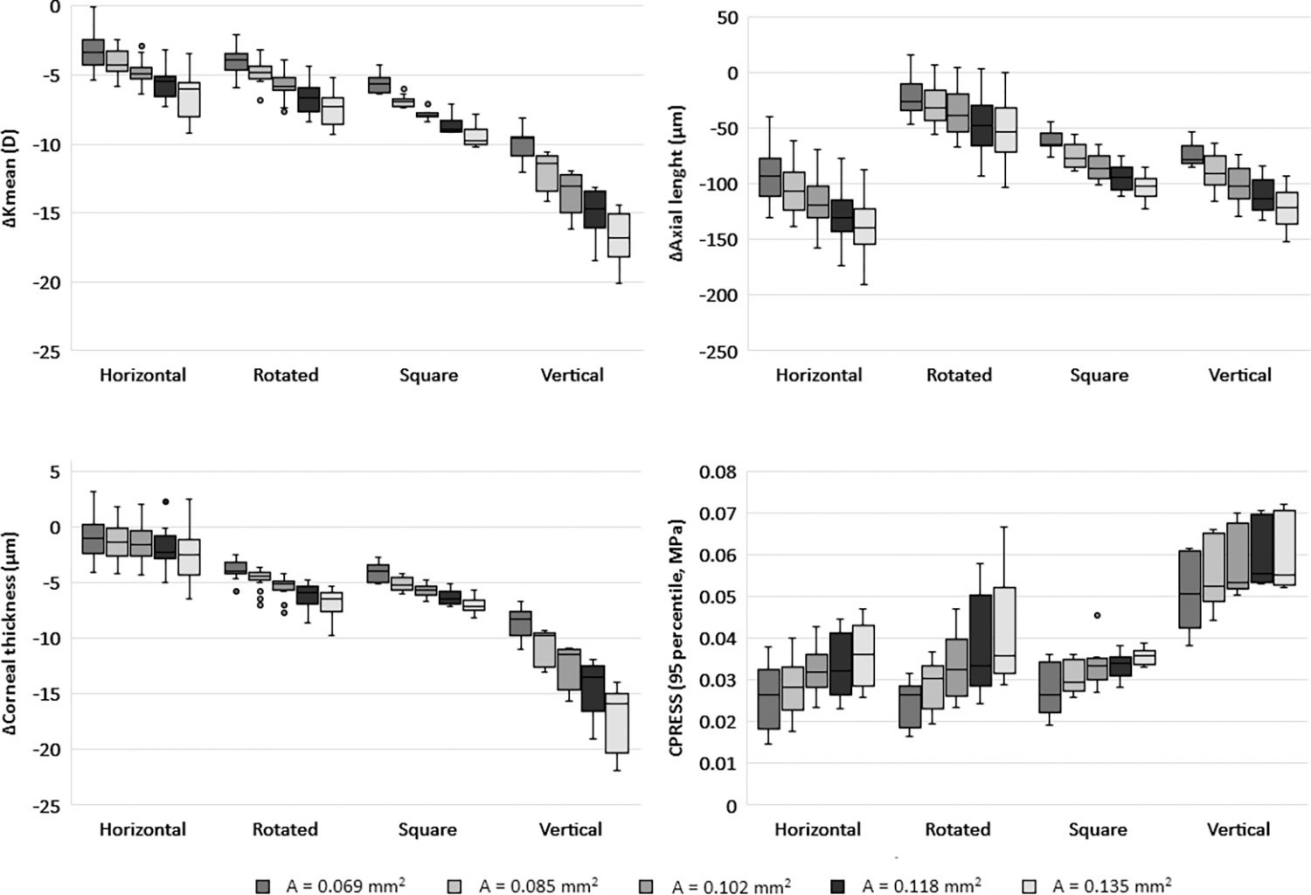
The change in K_mean_ (a), axial length (b), and corneal thickness (c) by ring category and cross-sectional area (indicated by A). Although ring category has a greater effect on these measures than the cross-sectional area, the area also affects the results: Increasing the cross-sectional area of the ring causes the cornea to flatten, resulting in a decrease in K_mean_ and axial length. The cornea also becomes thinner when larger rings are used and the contact pressure between the ring and the cornea increases. Within each ring category, the area significantly affects the outcomes (p < 0.043), except for the change in corneal thickness of the horizontal category (p > 0.197), and for the contact pressure of the vertical ring category (p > 0.164).

Similar to diameter, the cross-sectional area of the ring does not have as great an impact on postoperative parameters as ring category. However, within each ring category, the different ring sizes often result in statistically significant differences in measured outcomes (Fig 8). Our results show that increasing the cross-sectional area of the ring leads to a flattening of the cornea with a decrease in K_mean_ and axial length. The cornea also becomes thinner when larger rings are used and the contact pressure between the ring and the cornea increases.

**Fig 9:**
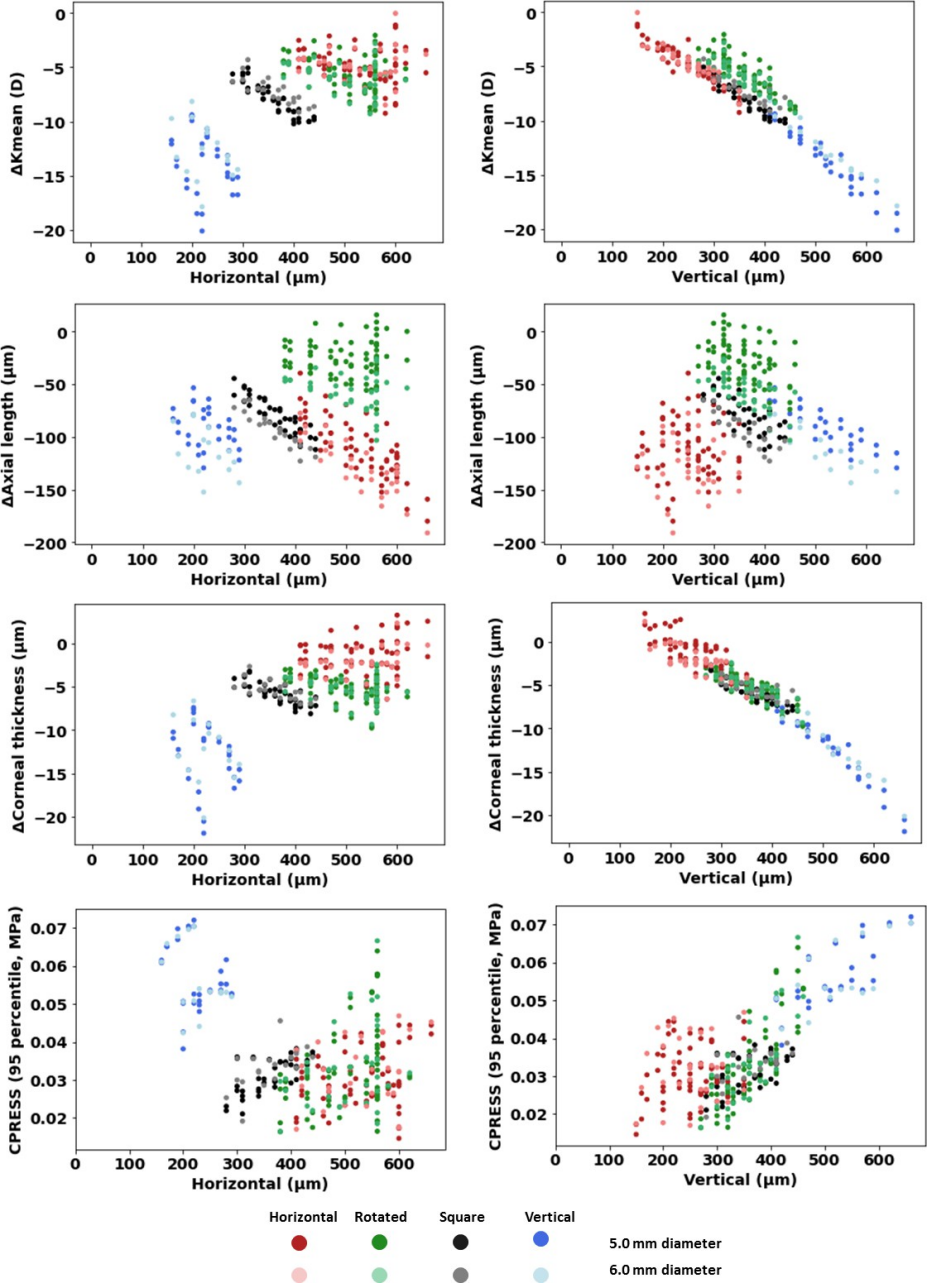
The change in K-means, axial length, and corneal thickness between treated and untreated corneas for all simulated rings (4 sizes for each of the 20 families) plotted as a function of ring dimensions: Horizontal, vertical, and diameter. Both the change in K_mean_ and axial length are well predicted by the vertical size of the ring, independent of the other design parameters or category. This figure includes the results of extremes of ICR’s optical diameter (5.0 and 6.0 mm).

The relationship between key clinical outcomes and ring design parameters was also examined for all categories combined (Fig 9). A strong monotonic relationship was found between the vertical dimension of the rings and the change in K_mean_ (ρ = -0.871) and corneal thickness (ρ = -0.955). The relationship appears to be general, as it crosses the boundaries of the different ring categories. For both strong relationships, an increase in the vertical dimension of the ring cross-section is correlated with a decrease in the change in K_mean_ and corneal thickness. A moderate positive monotonic relationship was also found between the change in maximum contact pressure and vertical dimension (ρ = 0.608), but there was no relevant relationship (ρ < 0.517) between these parameters and the horizontal dimension, diameter, or volume of the ring (Fig 9 and Table 2). Moreover, the change in axial length could not be related to any of the basic design parameters of the ring.

**Table 2:** Spearman’s rank correlation ρ (two-sided) between the basic parameters of the ring design (horizontal and vertical dimensions) and the mechanical and optical changes induced by implantation of the ring into the cornea.

	Horizontal		Vertical	
	p-value	ρ	p-value	ρ
**ΔK**_**mean**_	<0.001	0.**4**97	<0.001	**-0.871**
**ΔAxial length**	0.373	-0.0516	<0.001	0.158
**ΔCCT**	<0.001	0.517	<0.001	**-0.955**
**CPRESS**	<0.001	-0.267	<0.001	**0.608**

**Fig 10:**
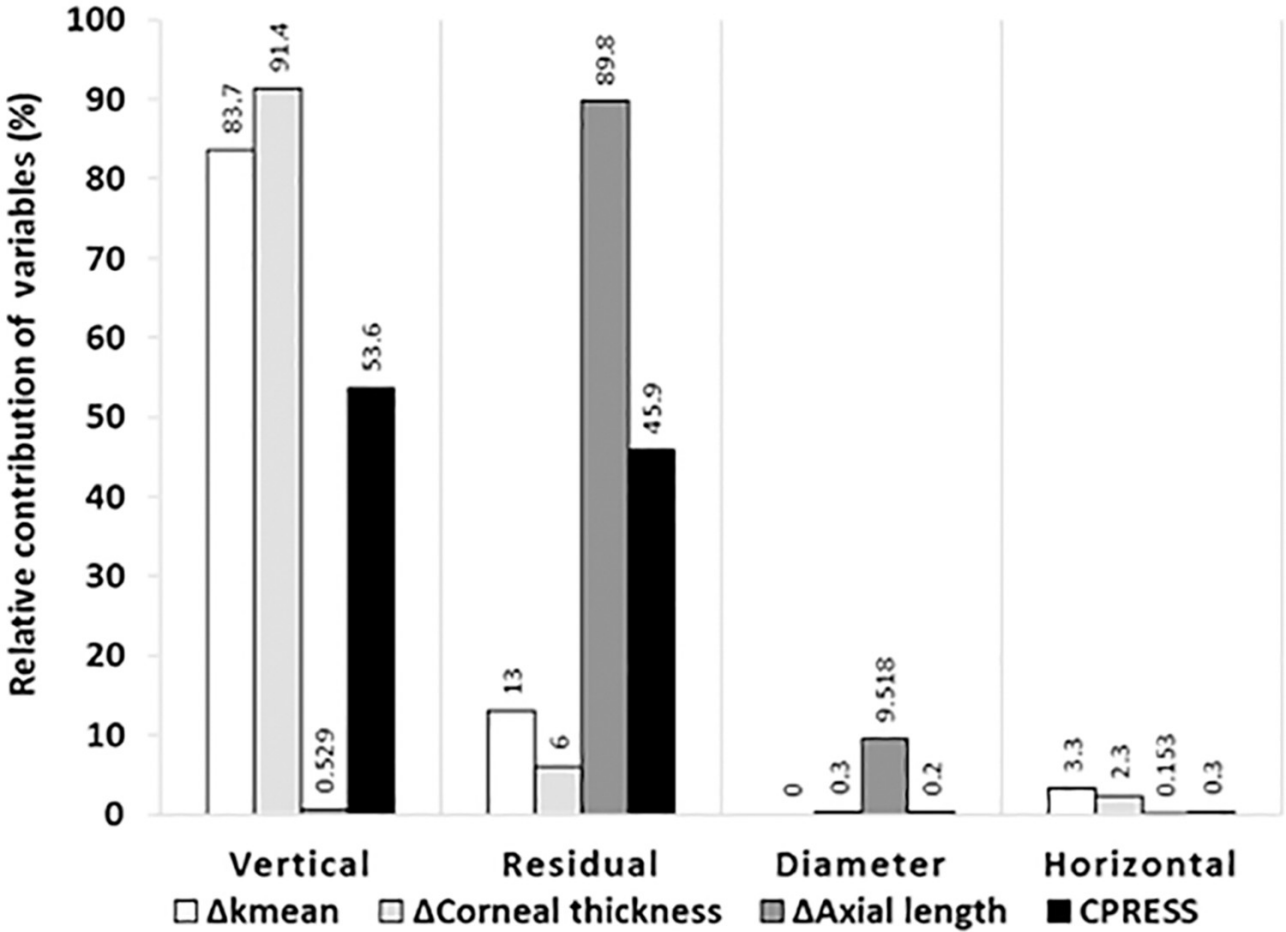
The relative contribution of each independent variable to the corneal mechanical and optical response. Both the vertical dimension of the implant and its design (i.e.: represented as residuals) contribute to the results, while the ring diameter and the horizontal dimension have only a marginal effect. While the vertical dimension contributes to about 90% of the induced K_mean_ and corneal thickness change, the implant design dominates the change in postoperative axial length. The vertical dimension and the implant design each contribute to about 50% of the corneal stress.

The vertical dimension of the ring explains about 90% of the change in K_mean_ and corneal thickness. Interestingly, the residual parameter, i.e., the design of the implant, explains only less than 15% of the optical results, but is mainly responsible for the change in axial length and contributes to about 50% of the contact pressure in the tissue. The implant diameter and the horizontal dimensions have only a minor influence on the results (Fig 10).

## Discussion

In this study, finite element simulations of ICR implantation were performed to evaluate the importance of ring design parameters on the visual and mechanical outcomes of the procedure. A total of 20 cross-sectional shapes in five sizes and three diameters were evaluated, resulting in 300 configurations. The change in K_mean_, axial length, corneal thickness, and pressure of the ring on the cornea were evaluated using automated finite element models.

The results showed that the rings effectively corrected corneal curvature. All configurations resulted in corneal flattening, associated with a slight posterior shift of the corneal region around the apex. However, the extent of correction depends on the implanted rings. Interestingly, the detailed design of the ring plays only a minor role in changing the corneal curvature. For this key optical parameter, the vertical size of the ring proved to be significant, being responsible for more than 80% of the change in K_mean_, 90% of the corneal thickness, and 50% of the contact pressure of the ring on the cornea. Our results also show that the horizontal size and diameter of the ring play only a minor role in the postoperative outcome.

These results showed that the primary parameter controlling postoperative anterior curvature is the vertical dimension of the ring, even before the ring diameter or the design of its cross-sectional shape. Therefore, this study supports Keraring’s approach to ring scaling, in which only the vertical dimension of the ring is changed while the horizontal size remains constant [31].

The effect of the detailed design of the ring is limited to the axial length and the contact pressure. The relationship between design and contact pressure can be explained by the fact that a smaller contact area results in a higher pressure for the same corneal deformation. Therefore, rings that provide greater contact with the stroma mechanically reduce pressure. The influence of the ring design on the axial length can be explained by the fact that the ring acts like a virtual limbus on the cornea around which the central part of the cornea rotates towards the posterior directions [23, 32]. Different ring designs lead to different rotation amounts and thus to different postoperative axial lengths.

Currently available surgical nomograms for implantable intracorneal rings (ICR) are based on qualitative observations and do not take into account the specific optomechanical response of the cornea to different segmental features [33]. These nomograms have limitations such as not conforming to mathematically predictable models and being subjective rather than objective [10]. A better understanding of the optical and geometric characterization of the cornea after ICR implantation, particularly based on measurable features, could lead to improved estimates of the postoperative corneal shape and more predictable refractive outcomes. The numerical approach proposed in this study allows for an objective selection of the implant to achieve the optimal optical correction, in addition to its ability to determine the most efficient implant design. In the future, these simulations can be extended to include patient-specific morphology to improve predictions. However, the determining parameter is the optical change induced by the ring.

In evaluating clinical outcomes after surgery, our calibrated in silico model showed changes in K_mean_ that were in good agreement with several clinical studies that showed postoperative changes between 4D and 8D and reported only minor differences between the effect of ring implantation in healthy subjects and keratocytes at the population level [16, 34–38]. Specifically for commercial rings, it has been reported that implantation of Kerarings in KC cornea causes an approximate change of 4.6D in K_mean_, which is close to the postoperative changes in K_mean_ of the horizontal rings in this study [39]. Similar changes in K_mean_ have also been reported for Myorings with different thicknesses and optical diameters [16].

The dependence of the results on the vertical and horizontal implant dimensions is also supported by clinical studies. Burrris et al. showed large changes in the flattening of the KC cornea-from 3.8D to 7.2D, when the ICRS thickness was increased from 0.26 mm to 0.45 mm [40], which is consistent with the strong dependence of refractive outcome on implant thickness in our study.

In addition, Alejandre et al [33] showed only a weak correlation between corneal flattening and horizontal implant size when implanting Keraring and Ferrara rings, which is consistent with the results of the present numerical study.

Unfortunately, clinical studies often report combinations of different ring typologies, corneal geometries, and degrees of pathology, making accurate comparisons with the numerical model difficult. In addition, published clinical studies typically provide limited information on patients’ corneal morphology, hindering the matching of specific rings to each patient and thus the replication of the study in silico. However, in a previous study, the authors validated the results of an in-silico model for a specific ring (MyoRing). By comparing the model predictions with clinical data from a cohort of patients with high myopia, we showed that the numerical model could accurately predict the change in spherical equivalent (SE) in treated patients, with an overall prediction error of less than 0.6 D. Based on these results, we assume that the validity of the numerical model also extends to different implant designs used in the present study. Our study has similar limitations to previous numerical studies of intrastromal corneal ring segments (ICRS), such as the use of continuous rings using axisymmetric simulations with average corneas or the use of mechanical models that are isotropic-hyperelastic [11, 22, 24, 41]. However, we go a step further by introducing a more robust numerical approach and focusing on testing different non-commercial configurations, which helps to understand the real impact of specific cross-sections on the corneal response after surgery.

A more accurate model that takes into account the 3D shape of the cornea would be needed to fully investigate the complex biomechanical deformations caused by ICR implantation in patients and their effects on patient vision. However, it should be noted that current nomograms do not take into account the 3D anatomy of the patient, so this information is not considered in clinical applications. However, the numerical approach developed in this study could be extended to a 3D model to investigate this issue in the future. In addition, other factors such as corneal mechanical properties, incision specification, tunnelling technique, and manufactured implant variability should be investigated in future research to provide a comprehensive evaluation of the outcome of the ring, taking into account patient and clinical variability. The study also induces a refractive error in a spherical model rather than correcting the refractive error of a myopic cornea. However, this technique allows evaluation of the optical change induced by implantation of the ring in a standardised model. Despite these limitations, the proposed numerical approach is able to capture the predominant effects of ring implantation, allowing the study of a large number of configurations in a reasonable amount of time.

In conclusion, this study has shown that the design of the ring plays an important role in the visual and mechanical outcomes of the ICR implantation procedure. The primary parameter controlling postoperative anterior curvature is the vertical dimension of the ring, ahead of the ring diameter or the design of its cross-sectional shape. These results support Keraring’s approach to ring scaling and can be used to improve surgical nomograms for ICR implantation.
